# Surgical treatment of tertiary hyperparathyroidism: does one fit for all?

**DOI:** 10.3389/fendo.2023.1226917

**Published:** 2023-11-02

**Authors:** Claudio Casella, Claudio Guarneri, Manuela Campanile, Xavier Adhoute, Pier Paolo Gelera, Riccardo Morandi

**Affiliations:** ^1^ Department of Molecular and Translational Medicine, Surgical Clinic, University of Brescia, Brescia, Italy; ^2^ Department of Clinical and Experimental Sciences, Surgical Clinic, University of Brescia, Brescia, Italy; ^3^ Service de Chirurgie Digestive et Endocrinienne, Hôpital Saint Joseph, Marseille, France; ^4^ Service d’Hépato-Gastro-Entérologie, Hôpital Saint Joseph, Marseille, France

**Keywords:** tertiary hyperparathyroidism, renal transplant, subtotal parathyroidectomy, total parathyroidectomy with autotransplant, hypercalcemia

## Abstract

**Background:**

Tertiary hyperparathyroidism (3HPT) is defined as a condition of excessive autonomous excretion of intact parathyroid hormone (iPTH) with persistent hypercalcemia (>10.5 mg/dL) that lasts for more than 12 months after a successful kidney transplantation, in the context of a long course secondary hyperparathyroidism (2HPT). The chronic high levels of iPTH cause a worsening of graft function, accompanied by systemic symptoms of hypercalcemia. The only curative therapy is parathyroidectomy (PTX). It remains unclear whether total parathyroidectomy with autotransplantation (TPTX-AT) or subtotal parathyroidectomy (SPTX) lead to better outcomes.

**Aims:**

The aim of this retrospective, single-institution cohort study is to evaluate the rate of persistent or recurrent disease and postoperative calcium/iPTH disturbances in patients treated with TPTX-AT or SPTX for 3HPT.

**Methods:**

A single-center retrospective analysis of 3HPT patients submitted to TPTX-AT or SPTX between 2007–2020 with at least 24 months follow-up was conducted. The outcome parameters included persistence/recurrence of disease, incidence of transitory hypocalcemia, and temporary/permanent hypoparathyroidism.

**Results:**

A cohort of 52 patients was analyzed and divided in two groups: 38 (73%) were submitted for TPTX-AT, and 14 patients (27%) were submitted for SPTX. The TPTX-AT population showed lower plasmatic calcium concentrations compared with the SPTX group during the entire follow-up period (p<0.001). There were eight cases (21%) of transitory hypocalcemia in the TPTX-AT group and none in the SPTX group, with p=0.065. Two cases (5%) of temporary hypoparathyroidism occurred in the TPTX-AT group and none in the SPTX group, with p= 0.530. There were no cases of permanent hypoparathyroidism and no cases of persistent disease. No statistical difference was assessed for the recurrence of 3HPT between the TPTX-AT group and the SPTX group (N=1, 3% vs N=1, 7%) (p=0.470).

**Conclusion:**

No significative difference was registered between the TPTX-AT and SPTX groups in terms of persistence/recurrence of disease, incidence of transitory hypocalcemia, and temporary/permanent hypoparathyroidism. Mean calcium levels iPTH values were statistically lower among the TPTX-AT group compared with the SPTX group while remaining always in the range of normality.

## Introduction

Tertiary hyperparathyroidism (3HPT) represents a pathological condition, usually subsequent to a long course secondary hyperparathyroidism (2HPT) that persists after successful renal transplantation (RTX). The incidence of 3HPT stands at about 20% in grafted patients and the need for surgical treatment occurs in 1–5% (1). The tertiary form represents 1.5–2% of all hyperparathyroidism cases; the female to male ratio is 4:1 and incidence peak occurs at 54 ± 15 years (2).

All 3HPT patients show persistent elevated plasmatic intact parathyroid hormone (iPTH) and plasmatic calcium (pCa) concentrations not explained by calcium carbonate or calcitriol supplementation (3). Nodular hyperplasia is the result of prolonged stimulation of parathyroid glands in chronic kidney disease (CKD) for hypocalcemia, low calcitriol values, and high levels of phosphate. Autonomization due to the nodular parathyroid degeneration causes iPTH oversecretion (4).

Patients with 3HPT usually complain of joint or bone pain, nephrolithiasis, vascular or soft tissue calcification, nephrocalcinosis, and fractures; gastro-duodenal ulcers, acute pancreatitis, and weight loss are also reported (5, 6).

Abnormally enlarged parathyroids can be detected with neck ultrasonography color Doppler imaging (US-CD) or sestamibi scanning (Tc-99m sestamibi SPECT-CT) evaluation. US-CD is affordable for high-weight glands (>1,000 mg), while sestamibi scanning is more reliable for smaller hyperplastic glands. Combination of the techniques allows more precise localization, even if in case of ectopic implants. In case of discordant results, four dimension computerized tomography (CT-4D) can be used (7).

Tertiary Hyperparathyroidism (HPT) pathogenic pathways and long-term implications in transplanted populations are not fully understood because of the lack of extended surgical series. However, a decreased renal function is known to be related to long course hypercalcemia and hypercalciuria with a higher risk of fractures and kidney failure (8–10).

Medical therapy with calcimimetics is used to control hypercalcemia but it is associated with low hyperparathyroidism (HPT) regression rates and poor amelioration of the low bone mineral density (BMD) linked to the increased risk of fractures. Nowadays, as reported in a recent review by Dulfer RR, parathyroidectomy (PTX) represents the definitive curative treatment of 3HPT with higher cure rates and lower complications compared with chronic medical therapy. PTX can indeed normalize hypercalcemia and hypophosphatemia and may have beneficial effects on BMD (11).

Indications for PTX are patients with hypercalcemia that persist after 12 months from the RTX, HPT-related clinical manifestations, or pathological bone pain/fractures due to severe osteopenia (12, 13). Although there is a lack of shared operative endpoints, an iPTH intraoperative drop >50% from the preoperative value can be considered the main goal.

Up to now, two surgical strategies are widely accepted: total parathyroidectomy with autotransplantation (TPTX-AT), which is resection of all four parathyroid glands and the normal parathyroid remnants’ implantation (30–50 mg) into the subcutaneous/muscular tissue of the forearm; and subtotal parathyroidectomy (SPTX), with the asportation of three parathyroid glands and 7/8 of the fourth one, that appears macroscopically normal,preserving 30–50 mg of well vascularized parenchyma (14).

No large data collection of 3HPT cases comparing the outcomes of TPTX-AT and SPTX are currently available. The aim of this retrospective, single-institution cohort study is to evaluate the rate of persistent or recurrent disease and postoperative calcium/iPTH disturbances in patients treated with TPTX-AT or SPTX for 3HPT.

## Materials and methods

We retrospectively analyzed 52 patients affected by 3HPT and treated with TPTX-AT or SPTX between 1 January 2007 and 31 December 2020 at our endocrine surgery center, with at least 24-months postoperative follow-up. Patients older than 18 years submitted to successful RTX with a previous diagnosis of 2HPT and who developed 3HPT with surgical indication for PTX were enrolled in the study. All cases had a history of hemodialysis (graft or tunneled catheter). No cases of peritoneal dialysis were registered. All patients were finally elected to surgery following a multidisciplinary discussion.

Hypercalcemia (pCa > 10.5 mg/dL), elevated iPTH values (normal range 14–65 pg/mL), or symptomatic calcium metabolism alterations (e.g., bone and joint pain, calciphylaxis, and osteodystrophy or pathological fractures) refractory to medical treatment represented our surgical indications. All patients with previous history of neck surgery for benign or malign thyroid/parathyroid disorders were excluded from the study architecture.

All patients were studied with preoperative US-CD or Tc-99m sestamibi SPECT-CT. All cases of unclear parathyroid localization were submitted to 4D-CT evaluation.

According to the literature, we define TPTX-AT as excision of all four parathyroid glands and the autotransplantation of 30–50 mg of normal-like parathyroid fragments into the subcutaneous forearm tissue. The asportation of three parathyroid glands and 7/8 of the most normal one preserving 30–50 mg of well vascularized parenchyma was defined as SPTX. Thymectomy was performed in each surgical treatment and any ectopic parathyroid tissue previously identified was removed. We decided to transplant the parathyroid remnants into the subcutaneous forearm tissue in order to perform an easier asportation in case of pathological degeneration (15). All parathyroids underwent histological evaluation; nodules’ dimension (length of major axis) and weight were reported.

All patients were accurately informed about the two different surgical approaches and their peculiarity in terms of perioperative and long terms management.

All patients received intravenous suppletive calcium therapy during hospitalization and oral calcium carbonate and calcitriol at discharge.

Diagnosis of persistent and recurrent disease was defined as iPTH > 300pg/mL (16) and/or PTH drop < 50% from preoperative level and/or pCa > 10.5 mg/dL within (persistent) or after (recurrent) 6 months from the PTX. Equally, diagnosis of temporary or permanent hypoparathyroidism was defined by the presence of an iPTH level < 13 pg/mL within or after 6 months. After surgery, all patients were treated with calcium supplementation for a minimum of 4 weeks; longer treatment was administered based on periodic plasmatic calcium re-evaluations. Transitory hypocalcemia was defined by postoperative pCa < 8.5 mg/dL with a resolution within 12 months.

Clinical serial follow-up evaluations were performed for a minimum of 24 months: iPTH and pCa assessment were registered each time.

### Statistical analyses

Data analysis and management were performed using IBM^®^ SPSS^®^ Statistics 20 for Windows^®^ software. A probability value of p < 0.05 was considered to be statistically significant. The normality of variables was tested using the Shapiro–Wilk method for normal distributions. All continuous variables were expressed as mean ± standard deviation, and categorical variables were expressed as numbers (percentage). Fisher’s exact test was used for the comparison of categorical data. The Mann–Whitney U test was performed to determine differences between groups.

## Results

### Population demographics

Between January 2007 and December 2020, a total of 52 patients were submitted to surgical treatment for 3HPT. The mean age was 53 ± 7 years and 26 (50%) were females. The mean time of hemodialysis before RTX was 61.30 ± 23.00 months and surgical treatment of 3HPT was performed after 56.56 ± 68.22 months from the RTX. Thirty-eight (73%) patients had TPTX-AT and 14 (27%) had SPTX. No concomitant thyroidectomies were performed among the two groups due to the absence of both preoperative and intraoperative indications.

Data of each group were collected and compared: no significant differences were registered in terms of sex, age, and time from RTX to PTX (p=0.071, p=0.378, p=0.06, respectively). The mean period of hemodialysis was longer among the TPTX-AT group (64.29 ± 26.29 months) compared with the SPTX group (53.21 ± 4.00 months), with p=0.016. In **Table 1**, the cohort’s demographic distribution is reported.

**Table 1:** Demographic characteristics.

	Total	TPTX-AT	SPTX	P Value
N (%)	52	38 (73)	14 (27)	
Age, y	53 ± 7	54 ± 7	50 ± 7	0.07
Sex, female (%) male (%)	26 (50) 26 (50)	20 (53) 18 (47)	6 (43) 8 (57)	0.38
Duration of dialysis (m)	61.3 ± 23	64.29 ± 26.29	53.21 ± 4	0.02
Time from Tx to PTX	56.56 ± 68.22	45.79 ± 62.11	85.79 ± 77.58	0.06

Data collection: Mean ± SD. Two-sided Chi-square or Student’s t test were used.

TPTX-AT, total parathyroidectomy with autotransplantation; SPTX, subtotal parathyroidectomy; Tx, renal transplantation; PTX, parathyroidectomy.

### Perioperative outcomes

Calcium assessment was evaluated before PTX and at patients’ discharge with no significant differences among the two groups (p=0.39, p=0.08), with the drop in percentages reported in **Table 2**. The mean preoperative iPTH value was higher in the TPTX-AT group (751.21 ± 678.24 pg/mL vs 455.43 ± 181.26), with p=0.018. In addition, the iPTH percentage drop at 10 minutes (’) after parathyroidectomy was greater among the TPTX-AT group (92.91 ± 5.02% vs 85.85 ± 5.95%; p<0.001). Coherently, iPTH values at 10 minutes (’) after parathyroidectomy were lower after TPTX-AT (35.08 ± 21.49 pg/mL vs 61.93± 27.07 pg/mL; p=0.003). In our cohort, supernumerary glands were found within the thymus in two cases, one in each study group, with an incidence of 7% in the SPTX group and of 3% in the TPTX-AT group (p=0.67). No significant differences were found in terms of histology, dimension, and weight between the two groups, as shown in **Table 2**.

**Table 2:** Perioperative results.

	TPTX-AT	SPTX	P Value
Preop pCa (mg/dL)	10.99 ± 0.50	11.12 ± 0.43	0.39
Preop iPTH (pg/mL)	751.21 ± 678.24	455.43 ± 181.26	0.02
Post 10’ iPTH (pg/mL)	35.08 ± 21.49	61.93 ± 27.07	<0.001
Post 10’ iPTH drop (%)	92.91 ± 5.02	85.85 ± 5.95	<0.001
Postop pCa (mg/dL)	8.68 ± 0.91	9.01 ± 0.40	0.08
Postop pCa drop (%)	20.91 ± 8.52	18.81 ± 5.24	<0.001
Histology Diffuse (%) Nodular (%)	4 (11) 34 (89)	4 (29) 10 (71)	0.12
Nodules dimension - major axis (cm)	1.30 ± 0.44	1.11 ± 0.51	0.18
Nodules weight (g)	1.21 ± 0.53	1.16 ± 0.47	0.27

Data collection: Mean ± SD or count (percentage). Two-sided Chi-square or Student’s t test were used.

TPTX-AT, total parathyroidectomy with autotransplantation; SPTX, subtotal parathyroidectomy; iPTH, intact parathyroid hormone.

### Postoperative and long-term follow up

**Figure 1:**
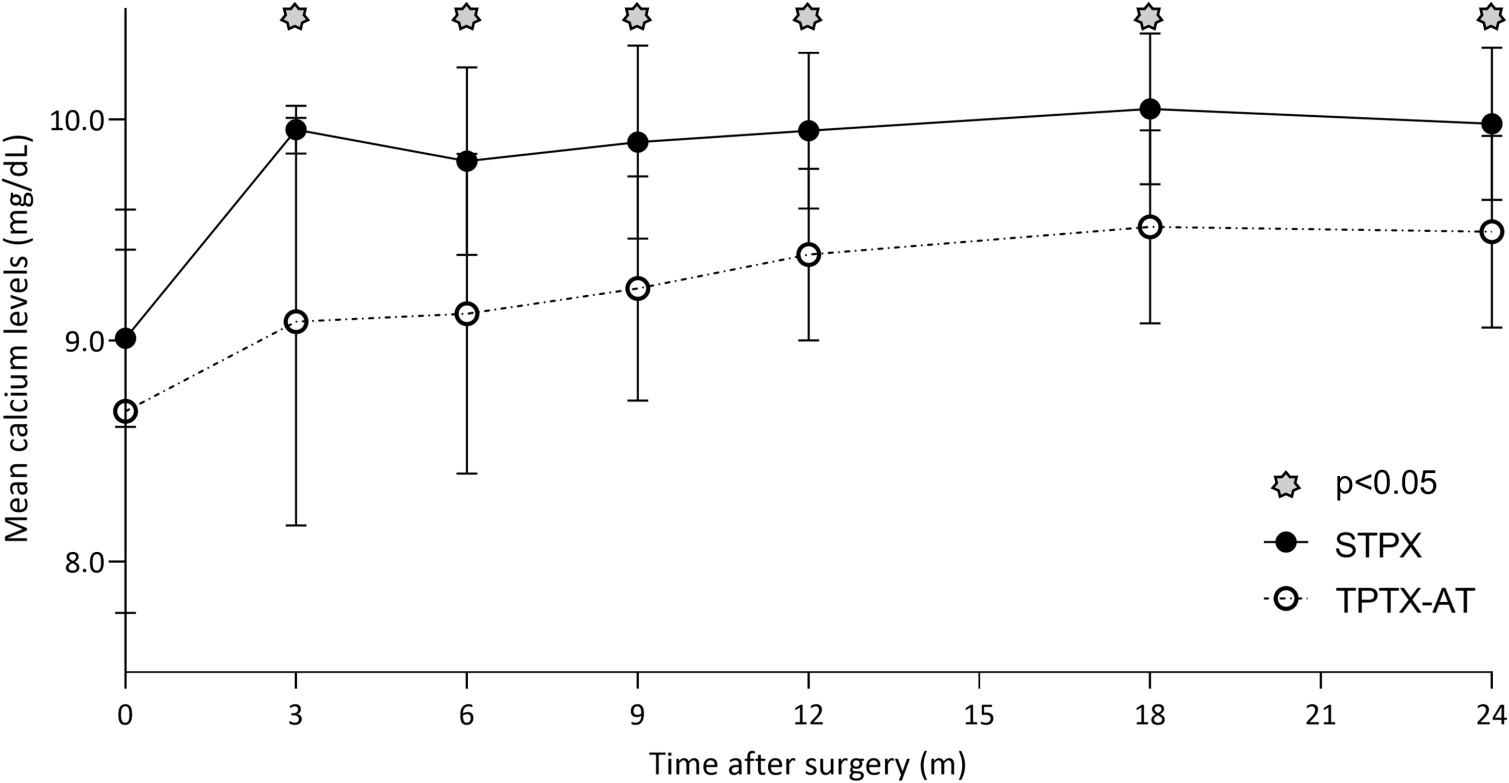
Although mean pCa levels were persistently inferior in the TPTX-AT group vs the SPTX group during the entire 24-month follow-up period, these values remained always in the range of normality.

All 52 patients had a complete 24-month follow-up data collection. The TPTX-AT population showed statistically significant lower pCa levels than the SPTX group during the entire 24-month (6-9-12-18-24 months) follow-up period (p<0.001), as reported in **Table 3** and **Figure 1**. On the other side, although iPTH levels were persistently inferior among the TPTX-AT group (**Table 3**), a significative p value was registered only at 6 and 9 months (p<0.001; p=0.031). Eight cases (21%) of transitory hypocalcemia were seen in the TPTX-AT group; none appeared in the SPTX group (0%), with a p value close to statistical significance (p=0.065). A higher 10 minutes (’) after parathyroidectomy iPTH percentage drop was evident in all these eight cases compared with the residual population values (95.59 ± 2.21% vs 90.18 ± 6.23%) with p<0.001.

**Table 3:** Long-term follow-up results.

	TPTX-AT	SPTX	P Value
6 months iPTH (pg/mL)	28.76 ± 9.57	48.64 ± 9.57	<0.001
6 months pCa (mg/dL)	9.12 ± 0.72	9.81 ± 0.42	<0.001
9 months iPTH (pg/mL)	33.29 ± 9.17	75.00 ± 64.33	0.03
9 months pCa (mg/dL)	9.24 ± 0.51	9.90 ± 0.44	<0.001
12 months iPTH (pg/mL)	42.77 ± 24.77	85. 36 ± 86.00	0.09
12 months pCa (mg/dL)	9.39 ± 0.39	9.95 ± 0.35	<0.001
18 months iPTH (pg/mL)	40.50 ± 27.11	83.71 ± 82.47	0.08
18 months pCa (mg/dL)	9.52 ± 0.44	10.05 ± 0.34	<0.001
24 months iPTH (pg/mL)	42.21 ± 27.84	88.00 ± 85.45	0.07
24 months pCa (mg/dL)	9.49 ± 0.43	9.98 ± 0.34	<0.001

Data collection: Mean ± SD. Two-sided paired t test was used.

TPTX-AT, total parathyroidectomy with autotransplantation; SPTX, subtotal parathyroidectomy; iPTH, intact parathyroid hormone.

Performing specific analysis of the population affected by temporary hypoparathyroidism or transient hypocalcemia, we found that patients with hypocalcemia had lower preoperative calcium levels (10.8 ± 0.4 mg/dL) compared with the rest of population (11.0 ± 0.5) with p<0.001. There was no statistic correlation between preoperative PTH levels and duration of dialysis. Concerning patients with temporary hypoparathyroidism, they were found to have lower preoperative PTH (439.0 ± 12.7 vs 778.1 ± 693.4; p=0.013). There was no statistic correlation between preoperative PTH levels and duration of dialysis.

At the end of the follow-up period, no cases of hypocalcemia were registered in both groups. Two cases (5%) of temporary hypoparathyroidism were found among the TPTX-AT population while no cases (0%) were registered in the SPTX group (p=0.530). Among both groups, there were no cases of permanent hypoparathyroidism. No cases of persistent disease were found in each cohort. No statistical difference was assessed for the recurrence of 3HPT between the TPTX-AT group and the SPTX group (N=1, 3% vs N=1, 7%) (p=0.470). In this context, particular interest is linked to the accuracy rates of localization preoperatory procedures, with the reference standard based on results of surgical exploration and histopathological examination. US-CD sensitivity was 80% while Tc-99m sestamibi SPECT-CT was able to localize pathologic glands in 82% of cases; 4D-CT was particularly useful to solve localization doubts, with a sensitivity of 83%.

The incidence of the three specific recurrence criteria among the two groups was not associated with a significant p value (**Table 4**).

**Table 4:** Focused outcomes.

	TPTX-AT	SPTX	P Value
Temporary hypoparathyroidism	2 (5)	0 (0)	0.53
Permanent hypoparathyroidism	0 (0)	0 (0)	/
Transient hypocalcemia (%)	8 (21)	0 (0)	0.07
6 months persistence 3HPT	0 (0)	0 (0)	/
24 months recurrence 3HPT (%) • iPTH > 300 pg/mL • pCa > 10.5 mg/dL • iPTH Drop < 50%	1 (3) • 0 (0) • 1 (3) • 1 (3)	1 (7) • 1 (7) • 1 (7) • 1 (7)	0.470 • 0.269 • 0.470 • 0.470

Data collection: count (percentage). Two-sided Chi-square was used.

TPTX-AT, total parathyroidectomy with autotransplantation; SPTX, subtotal parathyroidectomy; iPTH, intact parathyroid hormone; 3HPT, tertiary hyperparathyroidism.

**Figure 2:**
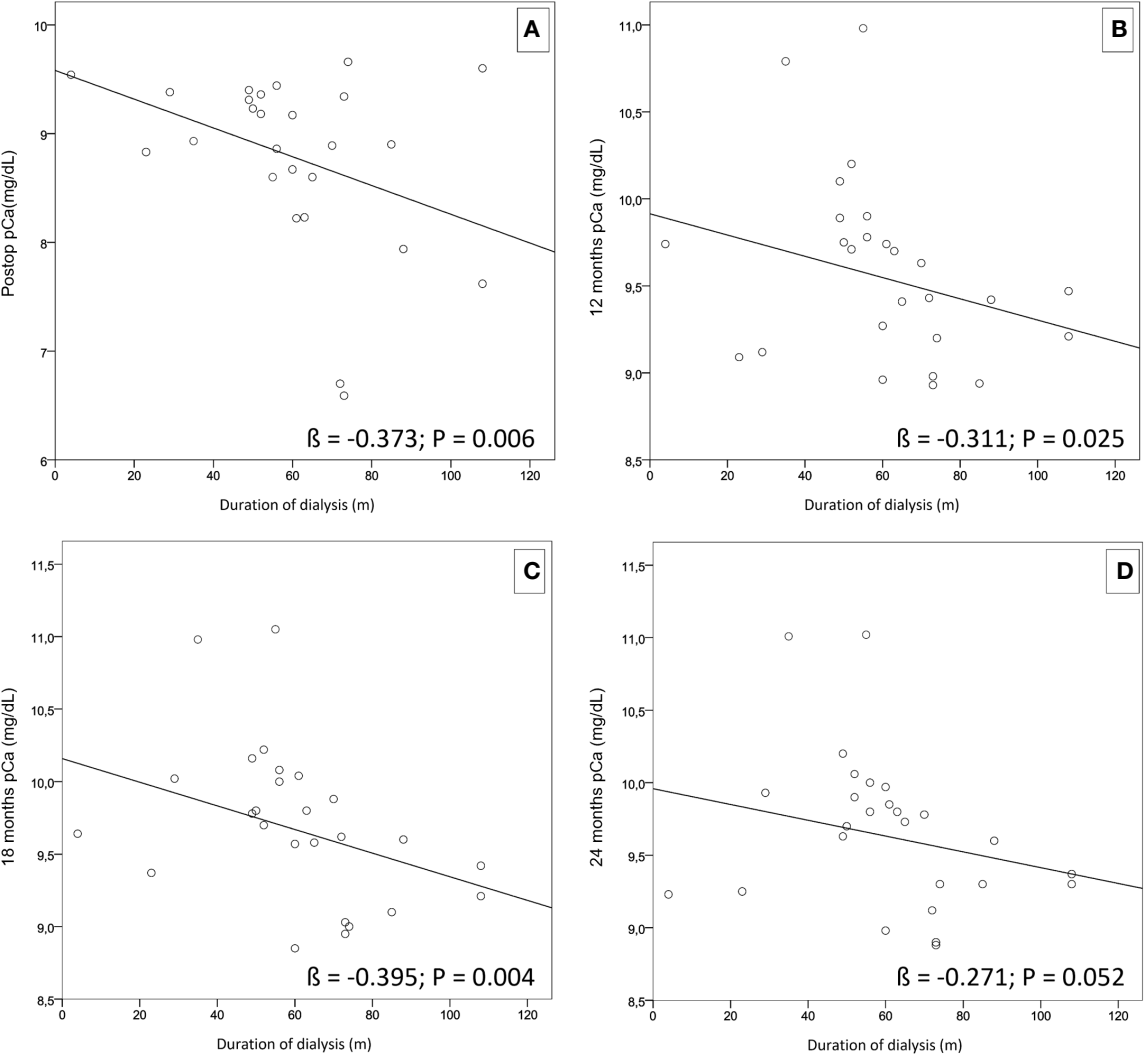
Simple linear regressions showing a negative correlation between the length of dialysis treatment and lower pCa levels during the follow-up period. **(A)** (ß = -0.373; p = 0.006). **(B)** (ß = -0.311; p = 0.025). **(C)** (ß = -0.395; p = 0.004). **(D)** (ß = -0.271; p = 0.052).

Furthermore, using a simple linear regression applied to our cohort, a negative correlation was found between length of dialysis treatment and lower pCa levels during the follow-up period (**Figure 2**). In addition, through multiple regression analysis, we found that the duration of dialysis did not impact the chosen surgical approach, related to pCa values at each follow-up re-evaluation (**Table 5**).

**Table 5:** Variable impact analysis.

	Duration of dialysis (m)	SPTX	TPTX-AT	P Value	P Interaction
Postop pCa (mg/dL)	≤ 60	9.14	9.19	0.697	0.885
	> 60	8.22	8.35	0.545	
12 months pCa (mg/dL)	≤ 60	9.98	9.52	0.014	0.695
	> 60	9.74	9.31	<0.001	
18 months pCa (mg/dL)	≤ 60	10.05	9.71	0.067	0.364
	> 60	10.04	9.39	<0.001	
24 months pCa (mg/dL)	≤ 60	10	9.67	0.076	0.909
	> 60	9.85	9.38	<0.001	

Data collection: Mean value. Multiple regression was used.

TPTX-AT, total parathyroidectomy with autotransplantation; SPTX, subtotal parathyroidectomy.

## Discussion

Nowadays, 3HPT remains an uncommon pathological condition that, after failure of medical therapy, requires surgery for definitive curative approach in patients with symptoms of hypercalcemia. Parathyroid asportation aims to normalize pCa and iPTH levels. Owing to the lack of multicentric randomized trials and the low incidence of disease, at this moment, there is no clear consensus about which of two most employed techniques (SPTX or TPTX-AT) allow better outcomes.

After a complete data assessment, we decided to stratify our population on laboratoristic findings registered preoperatively and during the whole follow-up period, primarily in order to investigate the rates of persistence/recurrence of disease after the two surgical treatments. Different outcomes are taken into consideration and often not all of them are included in the same study among the available literature. Thus, in this study, to be as inclusive as possible, the diagnosis of persistent and recurrent disease was defined considering multiple items: plasmatic iPTH concentration, percentage of iPTH drop, and pCa levels registered within or after 6 months from PTX.

Thanks to the analyses of the three items previously mentioned, we found no persistence of disease equally in both groups, demonstrating the superimposable rate of cure between the different surgical approaches.

According to Gasparri et al., 2001, our incidence of recurrent disease was 3% after TPTX-AT and 7% after SPTX, with no statistically significative differences obtained by the comparison of each of the three items (17). These outcomes could be potentially explained by the surgeon’s expertise in facing different preoperative and intraoperative situations and choosing the most suitable strategy to reach a superimposable disease-free result. According to the Miami Criteria, obtaining an iPTH drop >50% is capable of predicting postoperative eucalcemia (18). Furthermore, as explained by several authors, an intraoperative iPTH drop higher than 80% of the basal value can bring a dramatic reduction of recurrences (19).

It appears interesting that in our study the percentage drop obtained in both cohorts was much higher than 80% and this could be connected to our low recurrences rates. Moreover, even if no statistical differences were registered among the two groups in terms of recurrences, in our series it appears significant that the percentage drop in the TPTX-AT population is five percentage points higher than in the SPTX value.

Therefore, even if nowadays is not possible to define the best surgical approach by linking lower recurrences to an higher iPTH drop, it should be considered as fruitful field for future research.

The wide range of ectopic parathyroid localization is known to be the cause of a low curative rate and reappearance of disease; in particular, the thymus is one of the most frequent ectopic sites. In our surgical series, all patients were submitted additional contemporaneous thymectomy and this could be further linked to a good response in terms of cure and recurrence rates (20).

While some authors have documented that more frequent hypocalcemia is linked to TPTX-AT (21), TPTX-AT patients, the prevalence of transitory hypocalcemia appears to be tending towards statistical significance (21% vs 0%, p=0.065).

In this context, it appears interesting to underline that although mean pCa values were persistently inferior in the TPTX-AT group vs the SPTX group, these values remained always in the range of normality. It should be taken into consideration that, despite no critical differences being found between the two surgical techniques, TPTX-AT is linked to more extensive manipulation of parathyroid glands that are unbound from their anatomical peduncle of vascularization and thus transplanted to subcutaneous fat tissue. No immediate reprise of function and calcium homeostasis is guaranteed; indeed, in the literature many cases are reported of persistent hypoparathyroidism if inadequate rooting takes over.

Furthermore, as known, chronic kidney disease–mineral bone disorder (CKD-MBD) represents a condition that also affect patients with 3HPT. As reported by Wazeri et al., 2019 (6), the development of parathyroid hyperplasia is linked to specific alterations in bone metabolism; indeed, the progression to a nodular hyperplasia, starting from a diffuse form, is developed in a vitamin D hunger state appearance. In the context of end-stage CKD with no functioning kidney parenchyma, a continuous absorption of calcium from the bone, will cronically lead to cortical bone loss. In these patients, as explained by Cartwright et al., 2023 (22), in the postoperative period, the so-called “hungry bone syndrome” may set in and plasmatic calcium homeostasis may not be guaranteed because of the increased calcium absorption by bones.

Literature reports a higher incidence of hypoparathyroidism after TPTX-AT versus SPTX. It is confirmed even in our series in which two cases of hypoparathyroidism after TPTX-AT were described (5% vs 0%) (23).

Although a complete resolution of the hypoparathyroidism was assessed at 6-month evaluation, the average iPTH levels remained constantly lower at the intraoperative post-dissection dosage and at each re-evaluation measurement in the TPTX-AT group.

Surgical approaches in parathyroid disease underwent relevant modifications in the last few decades. Alongside the well-known total and subtotal parathyroidectomy, other “image-guided” approaches have been developed to allow a more limited glandular excision, for example, the so-called “less than subtotal parathyroidectomy” in which only glands with preoperative or intraoperative pathological features are removed (24).

In the literature, during the past two decades, several experiences of this technique have been reported, principally in the context of a primary hyperparathyroidism, with successful postoperative results that, unfortunately, did not seem to be obtained in the context of Tertiary hyperparathyroidism (THPT) conditions (25).

Indeed, despite apparently good PTH reduction in the immediate postoperative period, persistence and recurrence of disease appeared to be deeply influenced by the insufficient amount of tissue removal (26).

Furthermore, even in presence of macroscopically normal glandular tissue, hyperplastic features are present at histological evaluation, thus, THPT patients should not be eligible for limited resections in the context of an apparent normal glandular morphology in order to avoid rapid recurrences and difficult cervical re-explorations in case of graft deterioration (27).

Lastly, even if total parathyroidectomy without autotransplantation has been described, because of several cases of persistent hypoparathyroidism reported in our first surgical experiences and in the literature too, such procedure has not been performed at our center for 20 years.

As **Supplementary Material**, a brief literature comparison is shown in **Supplementary Table 2**.

## Conclusions

Although the shared indications for surgical treatment of 3HPT are well defined, no consensus has still been declared about the best surgical approach to PTX and which postoperative outcomes must be primarily taken into consideration.

Nowadays, in the context of 3HPT, the literature accepts different surgical management based on the surgeon’s choice and experience (28–33). The results of the postoperative outcomes described by our study embrace this state of the art, allowing well-trained endocrine surgeons to choose between TPTX-AT and SPTX to offer patients a case-specific tailored approach. Since just few cases of this rare condition are treated every year, a collegial expertise can be extremely useful in reaching the more correct work-up for each patient. Therefore, it is our belief that each surgical indication should include an adequate patient’s informed interview, a preoperative multidisciplinary staff discussion based on imaging evaluation, and the ability to calibrate the surgical approach considering the intraoperative findings.

The study could be limited by its retrospective monocentric nature, by the lack of randomization, and by the exiguity of sample size. On the other side, the main force of the project is represented by the standardized data collection and the long course follow-up.

The authors aim to encourage further studies on wider populations and cooperative data sharing in order to define the best surgical treatment.

## Data availability statement

The raw data used are available from the authors upon request by any qualified researcher.

## Ethics statement

According to current Italian Law, the approval of the Ethics Committee for a retrospective observational study is not required. Informed consent was obtained from all patients.

## Author contributions

CC, MC, XA, RM, and CG contributed to the conception and design of the work. RM and CG participated in data analysis and text editing. RM, CG, and PG participated in data collection and patients’ follow-up. CC, MC, and XA contributed to text revision and approved the final manuscript. All authors contributed to the article and approved the submitted version.

## Supplementary material

Supplementary Figure 1 Mean iPTH levels after surgery among the two populations. As shown, mean iPTH was lower in the STPX group and a p value < 0.05 was registered at patients’ discharge and at 6-9 months follow-up (asterisk labeled).

Supplementary Figure 2 Mean calcium levels at preoperative and postoperative (at time of patients’ discharge) evaluations. Preop pCa in the TPTX-AT group and the SPTX group (10.99 ± 0.50 vs 11.12 ± 0.43; p=0.39). Postop pCa in the TPTX-AT group and the SPTX group (8.68 ± 0.91 vs 9.01 ± 0.40; p=0.08).

Supplementary Figure 3 Mean iPTH levels at preoperative and postoperative (evaluated at 10’ after PTX) dosages. Preop iPTH in the TPTX-AT group and the SPTX group (751.21 ± 678.24 vs 455.43 ± 181.26; p=0.02). Post 10’ iPTH in the TPTX-AT group and the SPTX group (35.08 ± 21.49 vs 61.93 ± 27.07; p<0.001).
