# Supplementary figures and images for: Surgical treatment of tertiary hyperparathyroidism: does one fit for all?
Source: Front Endocrinol (Lausanne). 2023 Nov 2;14:1226917. doi: 10.3389/fendo.2023.1226917 (PMC10652876; doi:10.3389/fendo.2023.1226917)

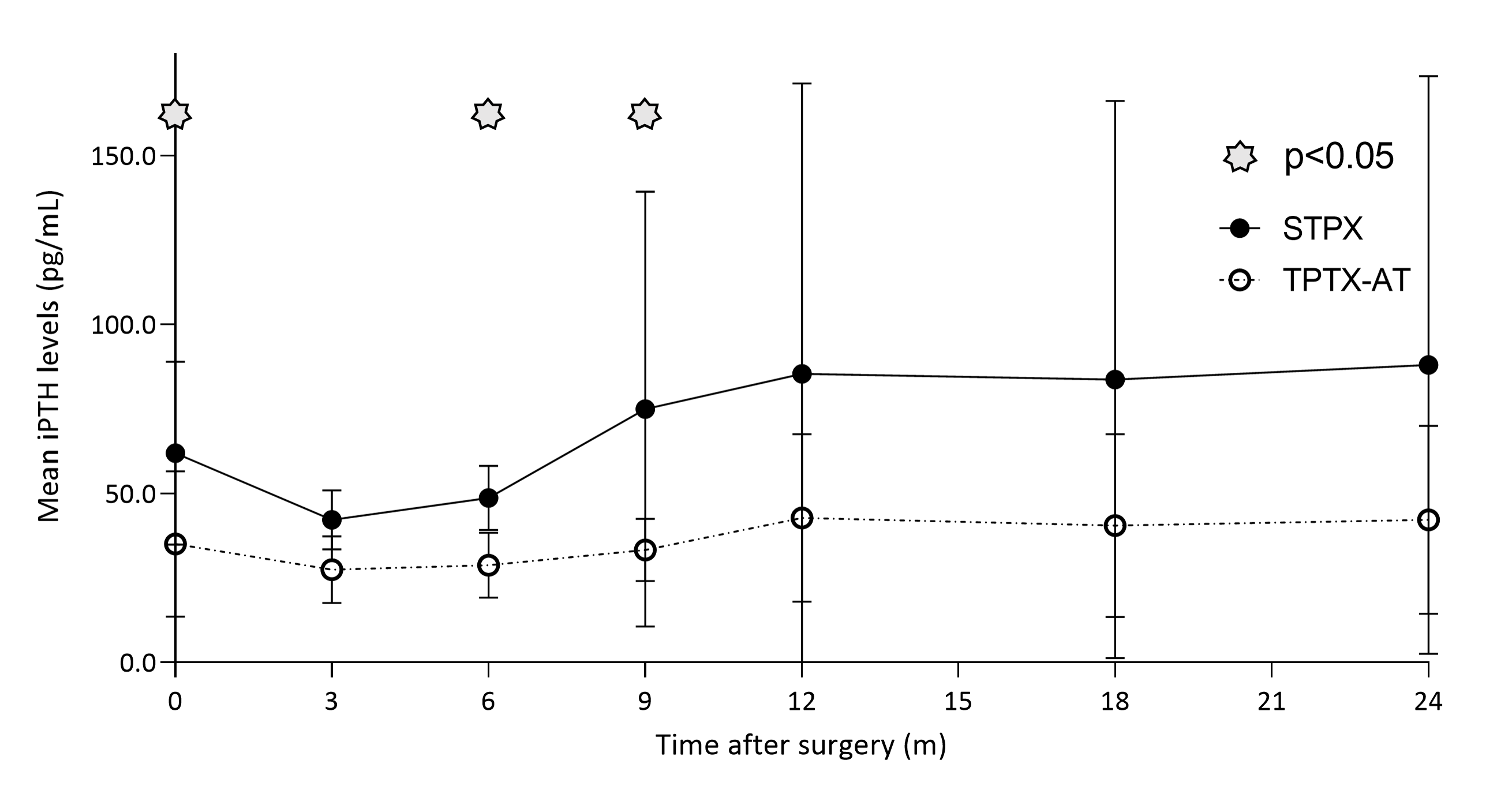

Supplement: Supplementary Figure 1 — Mean iPTH levels after surgery among the two populations. As shown, mean iPTH was lower in the STPX group and a p value < 0.05 was registered at patients’ discharge and at 6-9 months follow-up (asterisk labeled). [file Image_1.tif]

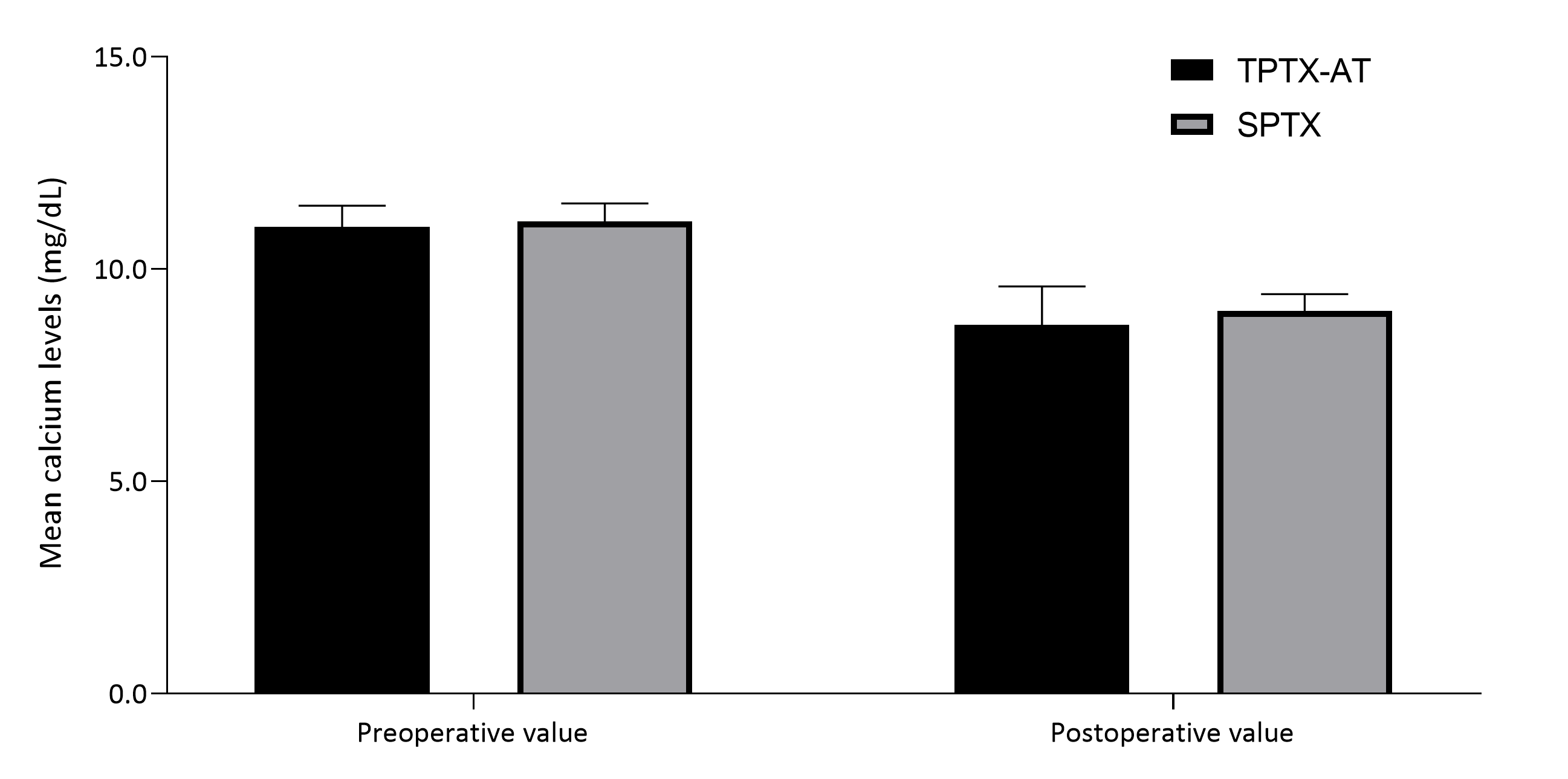

Supplement: Supplementary Figure 2 — Mean calcium levels at preoperative and postoperative (at time of patients’ discharge) evaluations. Preop pCa in the TPTX-AT group and the SPTX group (10.99 ± 0.50 vs 11.12 ± 0.43; p=0.39). Postop pCa in the TPTX-AT group and the SPTX group (8.68 ± 0.91 vs 9.01 ± 0.40; p=0.08). [file Image_2.tif]

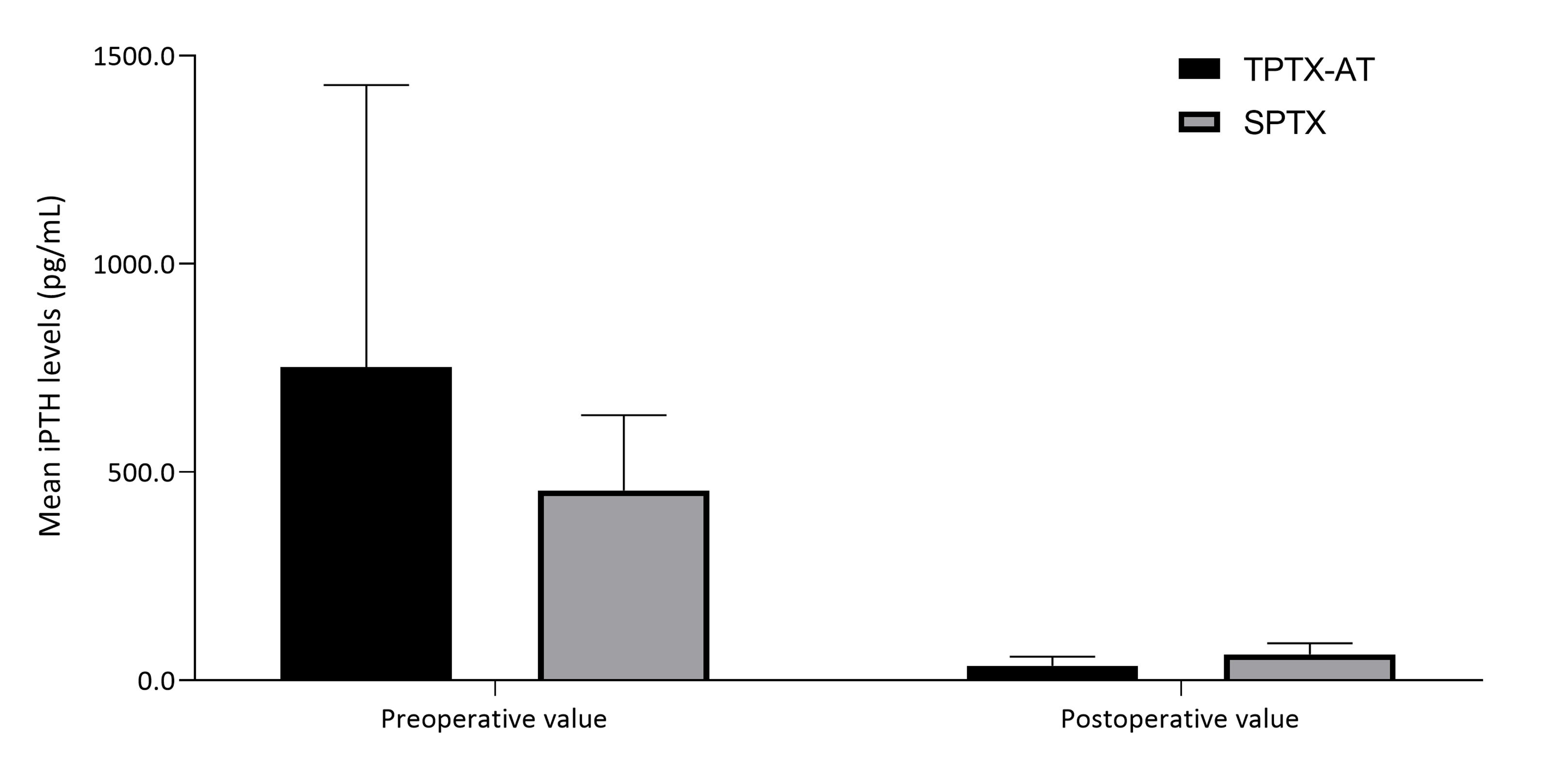

Supplement: Supplementary Figure 3 — Mean iPTH levels at preoperative and postoperative (evaluated at 10’ after PTX) dosages. Preop iPTH in the TPTX-AT group and the SPTX group (751.21 ± 678.24 vs 455.43 ± 181.26; p=0.02). Post 10’ iPTH in the TPTX-AT group and the SPTX group (35.08 ± 21.49 vs 61.93 ± 27.07; p<0.001). [file Image_3.tif]
