# Supplementary material for: Surgical treatment of tertiary hyperparathyroidism: does one fit for all?
Source: Front Endocrinol (Lausanne). 2023 Nov 2;14:1226917. doi: 10.3389/fendo.2023.1226917 (PMC10652876; doi:10.3389/fendo.2023.1226917)
Supplement: Supplementary file 4 [file DataSheet_1.docx]

**Supplementary Material**

**Table 6. (Supplementary Material)** Additional baseline characteristics

|  | TPTX-AT | SPTX | P Value |
| --- | --- | --- | --- |
| Plasmatic 25(OH)D3 levels (ng/mL) | 17.6 (13.2 – 24.5) | 17.9 (10.4 – 26.0) | 0.71 |
| Plasmatic creatinine levels (mg/dL) | 1.0 (0.6 – 1.5) | 1.2 (0.7 – 1.4) | 0.49 |
| Plasmatic phosphorus levels (mg/dL) | 2.7 (2.2 – 3.1) | 2.8 (2.4 – 3.7) | 0.28 |
| Calcimimetic treatment | 36 (95%) | 13 (93%) | 0.54 |
| Calcifediol treatment | 7 (18%) | 2 (14%) | 0.38 |
| Calcitriol treatment | 7 (18%) | 2 (14%) | 0.38 |
| T Score (lumbar spine) | -1.6 (-2.3 – -1.1) | -1.5 (-2.4 – -1.2) | 0.12 |
| Calciphylaxis | 0 (0%) | 0 (0%) | / |
| Hypertension | 29 (76%) | 11 (79%) | 0.23 |
| Renal stones | 5 (13%) | 1 (7%) | 0.62 |
| Cognitive disorder (asthenia, irritability, anxiety, poor concentration) | 21 (55%) | 8 (57%) | 0.31 |

Data collection: Median (min-max) or count (percentage). Two-sided Chi-square or Student’s t test were used.
TPTX-AT, total parathyroidectomy with autotransplantation; SPTX, subtotal parathyroidectomy.

**Table 7. (Supplementary Material)** Literature comparison

| Author | Year | Study design | Population | Suggested PTX |
| --- | --- | --- | --- | --- |
| Choi [28] | 2021 | MRS | 105 | SPTX |
| Sakman [29] | 2014 | SRS | 50 | TPTX-AT |
| Zmijewski [30] | 2019 | SRS | 46 | TPTX-AT |
| Hsieh [31] | 2012 | SRS | 14 | SPTX |
| Schlosser [32] | 2007 | SRS | 69 | SPTX |
| Sadideen [33] | 2011 | SRS | 26 | TPTX |
| Isaksson [34] | 2018 | ROS | 824 | TPTX |

MRS, multi-center retrospective study; SRS, single-center retrospective study; ROS, retrospective observational study; TPTX-AT, total parathyroidectomy with autotransplantation; SPTX, subtotal parathyroidectomy; TPTX, total parathyroidectomy.
